# Marked Cauda Equina Compression Secondary to Intervertebral Disc Protrusion Resulting in Severe Chronic Neuritis and Neural Fibrosis in a Dog

**DOI:** 10.1155/crve/3879955

**Published:** 2026-06-15

**Authors:** Eva Epstein, Allison Mooney

**Affiliations:** ^1^ Department of Neurology, West Vet Boise Animal Emergency & Specialty Care Center, Garden City, Idaho, USA

## Abstract

A 7‐year‐old male intact German shorthaired pointer was referred to a board‐certified veterinary neurologist for further evaluation of a chronic (> 6 months) history of gradually progressive paraparesis, limp tail, and spinal pain despite medical management of these clinical signs and presumptive neurologic disease with the family veterinarian. Radiographs revealed evidence of intervertebral disc space collapse, endplate sclerosis, and bridging spondylosis at L7‐S1, and the patient was presumptively diagnosed with degenerative lumbosacral stenosis (DLSS). Initial medical management included oral analgesics, oral anti‐inflammatories, lifestyle changes, and epidural steroid injections. Ultimately, progression of the patient′s clinical signs resulted in referral to a board‐certified veterinary neurologist. Magnetic resonance imaging revealed marked bilateral L7 nerve root enlargement with L7‐S1 foraminal widening and concurrent L7‐S1 intervertebral disc extrusion, causing moderate to severe left‐sided compression of the cauda equina. Given the marked changes of nerve roots on imaging and foraminal widening, consideration was given to a neoplastic process versus inflammatory neuritis. A lumbosacral dorsal laminectomy, partial discectomy, and left L7 nerve biopsy were performed. Histopathology of the L7 nerve root revealed chronic neural fibrosis with atrophy and mild neuritis, consistent with the chronic, progressive nature of the patient′s clinical signs and severe compression of the cauda equina. These dramatic and irreversible changes may have been avoidable or at least minimized with earlier diagnosis and aggressive surgical intervention, highlighting the importance of developing standardized, evidence‐based recommendations for management of dogs with DLSS similar to the framework that exists in human intervention for treatment of cauda equina syndrome (CES).

## 1. Introduction

Degenerative lumbosacral stenosis (DLSS) in canine patients is a term used to describe a variety of changes involving various structures of the lumbosacral (LS) junction, including intervertebral disc protrusion, endplate sclerosis, LS spondylosis, vertebral body misalignment, facet joint hypertrophy, and/or interarcuate ligament hypertrophy, resulting in compression of the cauda equina. One or more of these changes may be present, causing compression of the cauda equina, resulting in a wide range of clinical signs, such as pain during normal activity, inability to perform working tasks, and development of neurological deficits affecting the pelvic limbs, tail, bladder, and perineum [1]. The clinical manifestation of these abnormalities is secondary to compression of one or more of the nerve roots of the cauda equina [1–3]. Middle‐aged, medium‐ to large‐breed dogs are most often affected by DLSS, with German shepherd dogs and other working breeds being over‐represented [1, 3, 4].

Diagnosis of DLSS is often guided by a combination of physical exam findings, baseline systemic workup (bloodwork and urinalysis), advanced imaging (magnetic resonance imaging [MRI] and computed tomography), and cerebrospinal fluid analysis [2, 3]. Baseline systemic workup is recommended to evaluate patient candidacy prior to general anesthesia and as initial screening for comorbidities. Cerebrospinal fluid analysis is typically utilized selectively for cases in which a more thorough workup is necessary to rule out potential differentials. At this time, no definitive consensus on the treatment of DLSS is available, and because of the wide spectrum of clinical signs, treatment is often guided by the severity of clinical signs. Dogs with mild clinical signs may be effectively managed with conservative therapy, including activity modification, oral anti‐inflammatories and analgesics, and rehabilitation. Intradiscal and epidural injections have been described for patients with mild to moderate clinical signs; however, long‐term efficacy is unpredictable. Surgical intervention is recommended for dogs with neurologic deficits or discomfort that is refractory to conservative therapy; however, several surgical techniques exist, including stabilizing (distraction and fixation) and decompressive (dorsal laminectomy, foraminotomy, dorsal annulectomy, and nucleotomy) procedures [5]. There is no “one‐size‐fits‐all” recommendation, which can be a point of disagreement between clinicians when determining the appropriate technique(s), which is ultimately guided by the underlying cause(s) of DLSS, whether that be instability, compression, or a combination of both [1–3, 6].

While there is no general consensus on how dogs with DLSS should be managed, it is accepted that delayed surgical intervention for dogs with more advanced clinical signs, particularly urinary and fecal incontinence, may result in poor surgical outcomes and irreversible damage to the cauda equina [2].

This case outlines the diagnostic workup, attempted medical management, MRI, intraoperative, and histopathologic findings from a dog with cauda equina neuritis and neural fibrosis secondary to chronic, severe DLSS and associated LS intervertebral disc protrusion.

## 2. Case Presentation

A 7‐year‐old male intact German shorthaired pointer was referred to a board‐certified veterinary neurologist and neurosurgeon for further evaluation of a > 6‐month history of progressive paraparesis, a limp tail, and spinal pain.

Six months prior to presentation to the veterinary neurologist, the patient was initially evaluated by the family veterinarian for progressive pelvic limb weakness of approximately 3 months′ duration. At that time, LS pain and discomfort upon prostatic palpation were identified on physical examination, and a complete blood count (CBC), chemistry panel, and lumbar radiographs were recommended. No clinically significant findings were identified on the CBC or serum biochemistry panel. In‐house review of the radiographs was interpreted as LS instability with compression of L7‐S1 and suspected prostatomegaly. Oral analgesics (gabapentin and firocoxib), oral antibiotics (cephalexin), and activity restriction were recommended by the family veterinarian. Two weeks later, at a recheck evaluation, the owner described a lack of improvement, now with a limp tail, increased whining and cowering, inability to lift his leg for urination, and pain when posturing to defecate. Re‐evaluation identified pelvic limb proprioceptive ataxia and persistent prostatic discomfort. No additional investigation of the patient′s suspected prostatomegaly was recommended or pursued; however, referral for advanced imaging for further workup of suspected neurologic disease was recommended.

Around 1 month later, the patient presented to another general practitioner for a second opinion. During this visit, an official review of the previously obtained radiographs, with a boarded veterinary radiologist, was recommended. Following review, intervertebral disc space collapse, endplate sclerosis, and bridging spondylosis at L7‐S1 were identified, with no radiographic evidence of prostatomegaly. No additional imaging was recommended or pursued at this time. LS pain was appreciated on physical exam, and a series of three Depomedrol epidurals (1 mg/kg into the LS epidural space on Days 0, 15, and 45) was recommended. The epidural protocol was pursued and performed via landmark palpation and the hanging drop technique with no reported complications. An estimated 1 week of improvement in clinical signs was reported by the owner following each epidural; however, all previously reported clinical signs eventually reoccurred. Due to a lack of resolution in clinical signs, referral to a veterinary neurologist for advanced imaging was pursued.

Upon examination with a veterinary neurologist, the patient was ambulatory paraparetic with a proprioceptive ataxia and slightly dropped in his left hock. The patient′s tail carriage was low with decreased tone, but anal tone was normal. Severe discomfort was elicited upon palpation of the LS region. There was moderate to severe bilaterally symmetrical atrophy of both pelvic limbs. Additional diagnostics were recommended to further investigate the etiology of the patient′s clinical signs, assess candidacy for general anesthesia, and screen for potential comorbidities, including a CBC (clinically unremarkable), chemistry (mild cholestatic hepatopathy, ALP 447 U/L), urinalysis (clinically unremarkable), urine culture (no growth), thoracic radiographs (within normal limits), and cystatin B (< 5 ng/mL). MRI (1.5 T Siemens MRI) revealed marked bilateral L7 nerve root enlargement with L7‐S1 foraminal widening with concurrent L7‐S1 intervertebral disc extrusion causing moderate to severe left‐sided compression of the cauda equina (Figure 1). A cerebrospinal fluid sample, obtained via lumbar puncture, revealed changes consistent with iatrogenic blood contamination, but no clinically significant abnormalities (appearance: clear, colorless; protein: 64.6 mg/dL, RBC: 2929 cells/*μ*L, nucleated cell count: 9 cells/*μ*L) [7, 8]. Given the extent of the nerve root enlargement and concurrent foraminal widening present on MRI, primary consideration was given to a neoplastic process, such as lymphoma, although severe bilateral L7 neuritis secondary to intervertebral disc protrusion versus an immune‐mediated neuritis was also considered. Brucella testing was not performed, as clinical history and imaging findings were not consistent with disconspondylitis [9, 10]. An LS dorsal laminectomy was recommended to allow for decompression of the cauda equina and biopsy of an L7 nerve root for histopathologic evaluation to screen for evidence of neoplasia.

**Figure 1:**
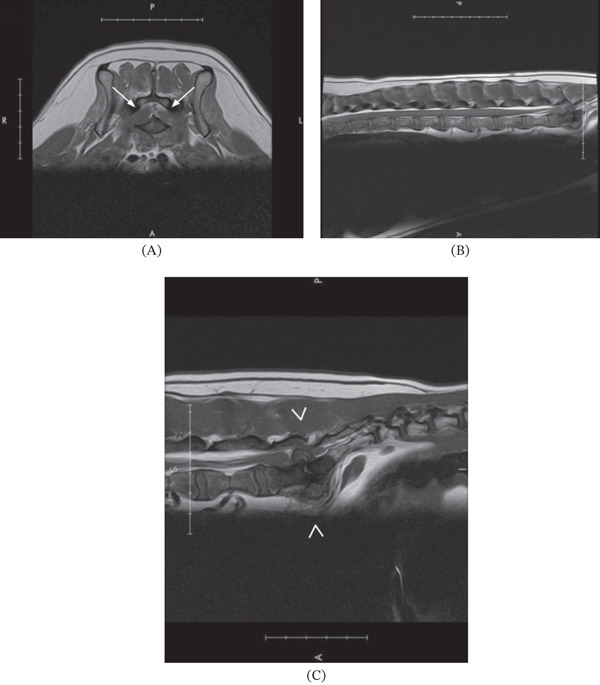
Pre‐ and postcontrast MRI images available, displaying (A) axial and (B, C) sagittal views of this patient′s caudal spine. Marked bilateral L7 nerve root enlargement (arrows) with L7‐S1 foraminal widening with concurrent L7‐S1 disc extrusion (arrowheads) is displayed, causing moderate to severe left‐sided compression of the cauda equina with no significant contrast‐enhancing lesions.

### 2.1. Surgery

Around 1 month following initial evaluation and workup with a veterinary neurologist, surgery was pursued. Preoperative physical and neurological evaluation was largely static compared to initial evaluation, but the patient was more comfortable following adjustments to his oral analgesic medications (transition from gabapentin to pregabalin, transition from acetaminophen to acetaminophen/codeine, addition of amantadine, and addition of a tapered course of prednisolone) and recommendations for strict activity restriction. An LS dorsal laminectomy and left L7 nerve biopsy were performed.

Intraoperatively, all visible nerve roots were described as markedly enlarged with an edematous appearance; however, they appeared normal in color. The enlargement was so profound that standard methods of retraction, such as utilizing the blunt, right‐angle end of a Senn retractor, were attempted but were unsuccessful at achieving adequate visualization of the dorsal annulus fibrosis of the L7‐S1 intervertebral disc. Therefore, a flattened Penrose drain was fed beneath different nerve roots using right‐angled forceps to allow for more gentle manipulation and retraction of the nerves to facilitate access to the L7‐S1 intervertebral disc space (Figure 2). A partial annulectomy was performed with serial incisions into the exposed L7‐S1 dorsal annulus with a Number 15 scalpel blade and curved mosquito hemostats. An incisional biopsy of the left L7 nerve root was performed by isolating an approximate one‐fifth diameter portion of the nerve root and creating a lengthwise incision with iris scissors in a caudal direction (moving in the same direction as the nerve fibers). An approximately 1‐cm‐long fascicular biopsy was obtained and submitted to an external reference lab for review by a boarded anatomic pathologist. The L7 nerve root was selected for biopsy due to accessibility. Given the concern for potential infiltrative neoplasia and the severity of the compression from the L7‐S1 intervertebral disc protrusion, stabilization was not considered a necessary component of providing relief of this patient′s clinical signs. There was no subluxation of S1 with the pelvic limbs in extension. Furthermore, given the fact that the L7‐S1 foramen was already massively expanded by the enlarged L7 nerve roots, truly being able to assess dynamic foraminal narrowing with a flexed view was determined to be unlikely. Ultimately, the radiologist and primary surgeon felt that given the amount of extruded disc material into the canal and the lack of sacral subluxation on extended views, the compression was most likely static in nature.

**Figure 2:**
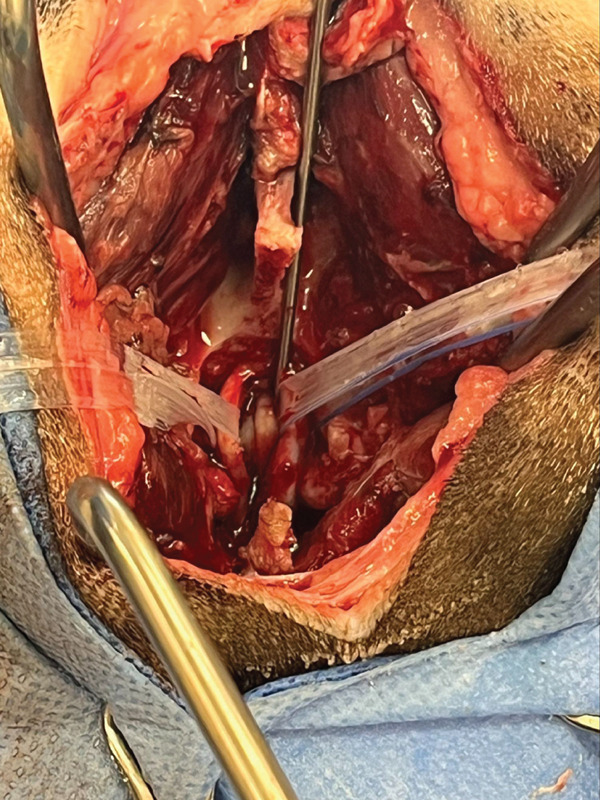
Intraoperative image displaying marked nerve root enlargement, requiring retraction to be performed with a Penrose drain.

### 2.2. Histopathology

Histopathology of the L7 nerve root revealed chronic neural fibrosis with atrophy and mild neuritis. These findings were characterized by the regional replacement of the nerve by fibrovascular tissue admixed with low numbers of macrophages, neutrophils, plasma cells, and lymphocytes. In fibrotic areas, axons are atrophied; no evidence of neoplasia was appreciated (Figure 3). These findings are consistent with the chronic, progressive nature of the patient′s clinical signs.

**Figure 3:**
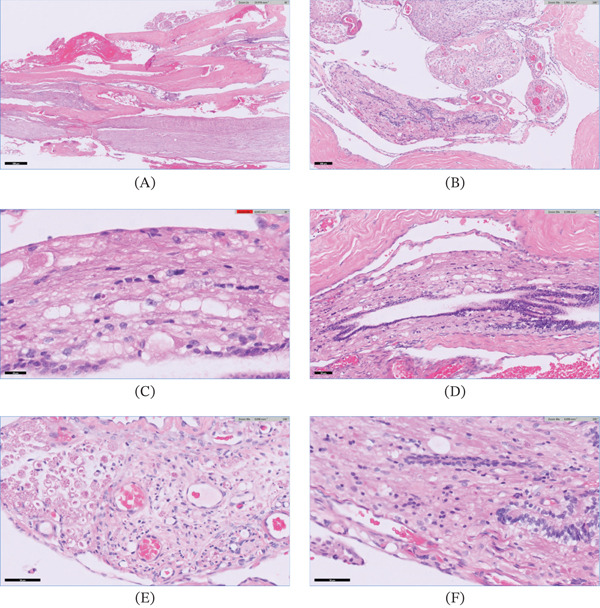
(A–F) Histopathology of the L7 nerve root revealed chronic neural fibrosis with atrophy and mild neuritis. These findings were characterized by the regional replacement of the nerve by fibrovascular tissue admixed with low numbers of macrophages, neutrophils, plasma cells, and lymphocytes. In fibrotic areas, axons are atrophied; no evidence of neoplasia was appreciated.

The L7‐S1 intervertebral disc was cultured intraoperatively out of an abundance of caution, given the previous steroid epidural injections, rather than out of a concern for discospondylitis, as the imaging findings were not consistent with this differential diagnosis. No organisms were isolated aerobically or anaerobically from the culture.

### 2.3. Postoperative Care

At‐home postoperative discomfort was managed with topical analgesics (fentanyl patch) and oral analgesics (pregabalin, amantadine, carprofen, and acetaminophen). Immediately postoperatively, the patient experienced gastrointestinal upset characterized by marked amounts of watery to blood‐tinged diarrhea, which was managed with a variety of supportive care medications. Six weeks of strict activity restriction, with a gradual increase in controlled activity at 4 weeks postoperatively pending surgical site healing and improvement of discomfort and neurologic status, was recommended. Physical rehabilitation was recommended but not pursued due to a lack of access to such services in the patient′s rural area. Trazodone was prescribed to help facilitate adequate postoperative rest, given the patient′s high energy and anxiety. The owner was educated on bladder expression techniques, and diazepam was provided for use as needed.

A combination of in‐person and telemedicine postoperative rechecks was scheduled at intervals of 2, 4, and 5 weeks postoperatively.

### 2.4. Outcome

Following his surgery, the formation of a large seroma occurred due to excessive activity as the patient continued to break out of his enclosure. This was resolved with warm compresses and adjustments to his living situation to help adhere to intensive activity restriction.

At approximately 2 weeks postoperatively, the patient was still experiencing occasional spastic urination and dropping of stool when barking. This change was investigated with the family veterinarian, and urinalysis and urine culture were performed (no significant findings). No treatment changes were made during this visit.

At his 4‐week postoperative recheck with the veterinary neurologist, the owner reported improvement in restlessness and discomfort. The pet continued to experience spastic urination and dropping of stool; however, no frequent dribbling of urine was noted. The owner reported that the patient would frequently posture to urinate (marking behavior), with only a small volume of urine being produced. On a recheck physical exam, he was strongly ambulatory with a mild paraparesis and mild proprioceptive ataxia and slightly decreased anal/tail tone. No discomfort could be elicited on LS palpation or bilateral hip extension. A bedside ultrasound of his urinary bladder was performed prior to and after urination at the examination, which revealed a moderately distended urinary bladder prior to micturition and a small, nearly empty urinary bladder following several markings in the hospital yard. His urinary and fecal incontinence was suspected secondary to neuropraxia from manipulation of nerve roots during surgery. These clinical signs could be a secondary complication of L7 nerve root biopsy; however, this is not supported by the absence of unilateral neurologic deficits. Tamsulosin and bethanechol were added to his treatment plan to help improve external urethral muscle relaxation and to facilitate bladder contractility, respectively.

At a final 9‐week postoperative telemedicine evaluation, the owner reports intermittent improvement in urinary issues. His family veterinarian continued to assess his bladder function, and the use of phenylpropanolamine was eventually substituted for other medications intended to improve bladder function at the discretion and recommendation of his family veterinarian. His comfort level was reported to have remained improved compared to his preoperative evaluation.

## 3. Discussion

In human medicine, cauda equina syndrome (CES) is a rare, serious condition characterized by dysfunction of nerve roots of the caudal equina, often secondary to lumbar disc herniation or trauma. Similar to canine patients with cauda equina compression secondary to DLSS, humans with CES can experience weakness, discomfort, and loss of sensation of the pelvic limbs, urinary and fecal incontinence, and lower back pain. In contrast to canine patients, a more in‐depth subcategorization system exists for CES, incomplete/partial CES (ICES), and complete/retention/incontinence CES (RCES), which are based on the severity of neurologic deficits. Patients with ICES typically experience motor weakness, sensory deficits, and urinary dysfunction, while patients with RCES typically experience more severe paralysis/plegia, more severe sensory loss, and urinary retention or incontinence. Regardless of categorization, this condition is often considered a surgical emergency, and prompt intervention is necessary to decrease the risk of permanent damage to the cauda equina and irreversible symptoms. [11]. Most studies have historically identified the most appropriate timing for surgical intervention for CES to be < 48 h for the best outcome, with surgery < 24 h not significantly affecting outcome for sphincter control [12]. However, a recent larger NICS study showed that surgery within 0–24 h of signs is associated with the best outcomes [13]. In contrast to human medicine, evidence‐based recommendations regarding the timing of intervention and the consequences of delayed treatment in dogs remain limited [1, 2, 6].

The patient described in this case report had exhibited progressive signs of CES for at least 3 months prior to the initiation of conservative therapy. By the time of evaluation with the neurologist, the patient had continued to experience neurological decline for an additional 6 months despite a course of epidural steroid injections. While conservative management is reasonably accepted for cases of presumptive or definitive DLSS, with mild clinical signs, pets with progressive clinical signs that are refractory to escalating treatment modalities warrant more extensive workup and treatment [2, 6]. In this case, the prolonged period between the start of his clinical signs and specialty referral for advanced imaging and decompressive surgery affected this patient′s outcome. For this reason, a categorization system comparable to that in humans with CES could help provide more specific treatment guidelines.

On MRI, the anatomical changes, particularly the nerve root enlargement and foraminal widening, were so striking that there was concern for infiltrative neoplasia, with less consideration given to chronic neuritis despite the presence of the concurrent LS intervertebral disc protrusion. Ultimately, neuritis and fibrosis of the left L7 nerve were diagnosed via histopathology and were attributed to the chronic, severe compression of the cauda equina secondary to the intervertebral disc protrusion [14]. While focal neuritis of the cauda equina has previously been described in a dog, this change has not previously been described secondary to severe DLSS [15]. These irreversible changes may have been avoidable or at least minimized with earlier diagnosis and aggressive surgical intervention, highlighting the importance of developing standardized, evidence‐based recommendations for management of dogs with DLSS similar to the framework that exists in human intervention for treatment of CES.

## Funding

No funding was received for this manuscript.

## Consent

No written consent has been obtained from patients, as no patient‐identifiable data has been included in this case report.

## Conflicts of Interest

The authors declare no conflicts of interest.

## Data Availability

Data sharing is not applicable in this article as no datasets were generated or analyzed during the current study.
